# Problematic Smartphone Use: Investigating Contemporary Experiences Using a Convergent Design

**DOI:** 10.3390/ijerph15010142

**Published:** 2018-01-16

**Authors:** Daria J. Kuss, Lydia Harkin, Eiman Kanjo, Joel Billieux

**Affiliations:** 1 International Gaming Research Unit, Psychology Department, Nottingham Trent University, Nottingham NG1 4FQ, UK; lydia.harkin02@ntu.ac.uk; 2 Computing and Technology Department, Nottingham Trent University, Nottingham NG1 4FQ, UK; eiman.kanjo@ntu.ac.uk; 3 Addictive and Compulsive Behaviour Lab., Institute for Health and Behaviour, Integrative Research Unit on Social and Individual Development (INSIDE), University of Luxembourg, Esch-sur-Alzette, L-4365 Luxembourg, Luxembourg; joel.billieux@uni.lu; 4 Addiction Division, Department of Mental Health and Psychiatry, University Hospitals of Geneva, 44041 Geneva, Switzerland

**Keywords:** smartphone, problematic mobile phone use, convergent design, focus group, survey

## Abstract

Internet-enabled smartphones are increasingly ubiquitous in the Western world. Research suggests a number of problems can result from mobile phone overuse, including dependence, dangerous and prohibited use. For over a decade, this has been measured by the Problematic Mobile Phone Use Questionnaire (PMPU-Q). Given the rapid developments in mobile technologies, changes of use patterns and possible problematic and addictive use, the aim of the present study was to investigate and validate an updated contemporary version of the PMPU-Q (PMPU-Q-R). A mixed methods convergent design was employed, including a psychometric survey (*N* = 512) alongside qualitative focus groups (*N* = 21), to elicit experiences and perceptions of problematic smartphone use. The results suggest the PMPU-Q-R factor structure can be updated to include smartphone dependence, dangerous driving, and antisocial smartphone use factors. Theories of problematic mobile phone use require consideration of the ubiquity and indispensability of smartphones in the present day and age, particularly regarding use whilst driving and in social interactions.

## 1. Introduction

The Western world has seen a significant increase in mobile technology use in the last decade. In 2016, the communications regulator Ofcom [1] referred to the UK as a “smartphone society”; 93% of the population own a smartphone, and users spend more time accessing the Internet via a phone than through other devices, such as laptops and desktop-computers. These recent trends suggest mobiles and the Internet have become intimately intertwined to enable “on-the-go” access to a range of facilities, including web-browsing, communication, shopping, banking, and gaming [1].

Recent research suggests a number of problems can result from smartphone overuse, including addiction-like symptoms and feelings of dependence [2,3], dangerous use, particularly whilst driving [4,5], and forbidden or prohibited use in areas such as libraries, classrooms, or public transport [6]. Accumulating evidence also connects excessive mobile phone use with increasing psychopathological symptoms, such as those related to depression and anxiety [7]. In other words, research suggests excessive mobile phone use can result from psychopathology and constitute a dysfunctional strategy to cope with adverse emotions. Similarly, King et al. [8] suggested that mobile phone checking can constitute a safety behaviour in anxious individuals. Internet-enabled devices may encourage checking behaviours by hosting a range of applications (or apps) with regular updates and notifications. Thus, mobile Internet use may increase habitual checking behaviours, which may contribute to developing and maintaining symptoms of psychopathology, such as addictive use [9]. Consequently, a growing number of studies are conducted to determine whether smartphone overuse constitutes a genuine addictive disorder (e.g., [10]), which is in line with the inclusion of a behavioural addiction category in the latest edition of the Diagnostic and Statistical Manual of Mental Disorders (DSM-5; [11]). Yet, to date, the evidence supporting problematic smartphone use as an addictive disorder is scarce, and the studies emphasizing behavioural and neurobiological similarities between problematic smartphone use and other types of recognised addictive disorders are limited [12].

### 1.1. Gaining a Contemporary View of Smartphone Behaviours

Past research on problematic mobile phone and addictive smartphone behaviours employed quantitative methodologies to examine negative consequences associated with smartphone use. Various ways of measuring problematic smartphone use have been proposed considering different criteria and sources, including empirical evidence [13,14], substance abuse criteria [4,15,16,17,18,19,20], pathological gambling criteria [19,21], reviews of the relevant literature [2,17,18,22,23,24], or Internet addiction criteria [23]. When it comes to determining when smartphone use becomes problematic, it is important to be aware that time spent using these devices is not a sufficient indicator. For instance, it has been found that time spent socialising on mobile apps left users with positive mood [25]. Thus, the types of smartphone interactions appear to have varying impacts on user wellbeing. However, merely reading, removing, and scrolling through messages leaves users with negative emotions [25]. In addition to utilising a quantitative research approach, an experiential perspective based on users’ own perceptions and understanding of their smartphone use may offer significant insights into what constitutes problematic smartphone use and how it is experienced on an individual level. User perceptions of smartphones can help to define what aspects of this technology are beneficial or problematic.

However, experiential evidence of mobile devices is outdated. Surveys capturing smartphone perspectives have failed to keep up with the speed of technological advancement and often do not reflect the full range of behaviours possible on modern smartphones [25]. Relatively recent smartphone interactions, particularly those which are supported by ‘on-the -go’ Internet technology, have not been accounted for and may influence problematic smartphone experiences. An experiential perspective based on users’ own perceptions and understanding of their smartphone use may offer significant insights into what constitutes problematic use and how it is experienced on an individual level. The present research aims to fill this gap in knowledge by using a mixed methods convergent design incorporating a qualitative exploration of perspectives on contemporary smartphone use.

### 1.2. Existing Measures of Problematic Smartphone Use

A theory of problematic mobile phone use [12] suggests that there are three pathways which may result in negative and pathological smartphone behaviours, namely (i) the excessive reassurance pathway, (ii) the impulsive-antisocial pathway, and (iii) an extraversion pathway. These pathways suggest that personality, psychopathological symptoms, and frequency of smartphone use can have particular problematic consequences. The Problematic Mobile Phone Use Questionnaire (PMPU-Q) [2] was developed to assess various facets of problematic mobile phone use. The original questionnaire included four subscales: (1) prohibited use; (2) dangerous use; (3) dependent use, and (4) financial problems resulting from use.

Contemporary publications and theoretical reflections on problematic smartphone use take different perspectives relative to Billieux et al.’s proposed model [12]. For instance, financial implications may no longer be considered a contemporary problematic use of smartphones. Recent evidence links the evolution of mobile phones to smart technology with many benefits; social applications such as WhatsApp and Skype can now facilitate communication with little cost to the user, and apps are available, which support financial and banking activities [26,27]. In addition, the US Department of Transportation reported smartphone technology as a key distractor which can deflect the attention of pedestrians and drivers, leading to potential collisions [28]. Considering this, previous survey measures of problematic behaviours excluding such contemporary activities may only partially record problematic experiences. Given the rapid developments in mobile technologies, changes of use patterns and possible problematic and addictive use, the aim of the present study was to test and validate an updated contemporary version of the original PMPU-Q using a rigorous and innovative convergent parallel design. In order to investigate the efficacy of the existing measure of these phenomena, a psychometric survey was included in this study which featured the PMPU-Q and validated measures of smartphone affect.

## 2. Methods

### 2.1. Design

**Figure 1:**
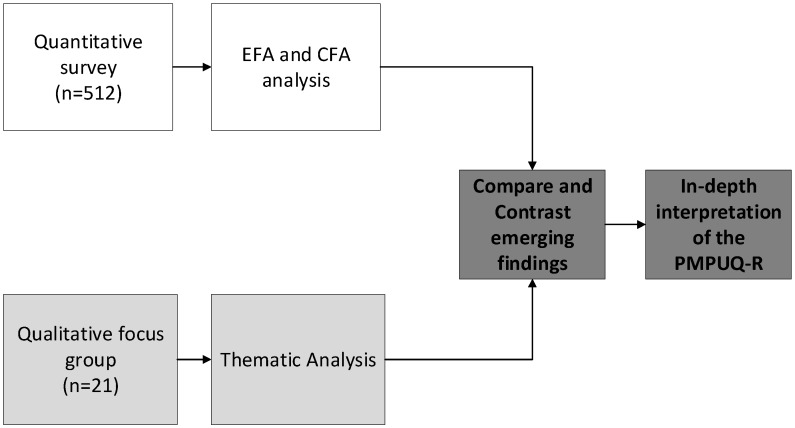
Convergent study design.

This study used a mixed methods methodological approach with a convergent parallel design. A mixed methods approach allowed for bridging two research traditions regarding problematic smartphone use by means of integrating large-scale psychometric inquiry with a qualitative analysis on personalized experiences, allowing for a better understanding of the validity of the PMPU-Q-R. Fetters et al. [29] identified a convergent parallel design as a suitable mixed method for investigating the validity of quantitative measures. Design convergence in this case refers to decreasing PMPU-Q measurement uncertainty by using different methods [29]. The updated PMPU-Q (PMPU-Q-R) was administered to a sample of smartphone users, together with a number of relevant other validated psychometric measures, to determine the construct validity and internal consistency of the measure. In the second phase, perceptions and experiences of smartphone use, including the respective usages (i.e., dependent, dangerous and prohibited), were explored using focus groups. This concurrent procedure was time efficient, and meant that interpretive analysis of each individual dataset informed the other [29]. This was important in the present study as interpretation required innovative, evidence-based reconceptualising of an evolving technology. Figure 1 demonstrates the convergent design employed in this study.

### 2.2. Study Recruitment

Smartphone users were recruited to the quantitative survey during December 2016 to March 2017 using opportunity and snowball sampling. Study advertisements encouraged smartphone users to follow a weblink to the survey hosted through Qualtrics in the UK. Offline advertisements were posted throughout university networks, and online advertisements were shared within student portals and social media networks, which focused on smartphone use. This social media dissemination included forums, Twitter, Facebook, and Reddit networks. Participants were considered eligible if they were smartphone users. Focus group participants were obtained from an opportunity sample of survey participants.

### 2.3. Study Procedures

#### 2.3.1. The PMPU-Q

The PMPU-Q was originally developed by Billieux and colleagues [2] and investigated four dimensions of problematic smartphone use: (1) prohibited use, (2) dangerous use, (3) dependent use, and (4) financial problems resulting from use. In this study, the financial problem scale was excluded as financial implications are no longer considered a contemporary problematic smartphone use [26,27]. In addition, items concerning pedestrian safety were included as dangerous items in the adapted PMPU-Q, as seen in Table 1. These adaptations produced the PMPU-Q-R, which was administered as a 17-item questionnaire. Responses were measured on a four-point Likert scale (ranging from 1 = strongly disagree to 4 = strongly agree).

#### 2.3.2. Validated Measures for Comparative Analysis

1. Smartphone Addiction and Social Media Disorder

This survey included the Smartphone Addiction Scale (SAS) [16] and the Social Media Disorder Scale (SMD) [30]. These two scales measure excessive smartphone use as an addictive behaviour, and thus included items adapted from the substance abuse literature. The SAS consisted of ten items assessing symptoms of smartphone addiction. Statements relating to smartphone addiction were rated on a seven-point Likert scale ranging from 1 = strongly disagree to 7 = strongly agree. The SAS has previously demonstrated good internal consistency and concurrent validity [16]. Cronbach’s alpha for this scale in the present study indicated good reliability (α = 0.88). Recent research indicates a strong association between social media and smartphone use [31], supporting the inclusion of a psychometric tool assessing social media addiction. The SMD scale consisted of nine items representing eight aspects of social media disorder: preoccupation, tolerance, withdrawal, displacement, escape, problems, deception, displacement, and conflict. Participants were asked to rate, on a five-point Likert scale, how often they had experienced a symptom of social media disorder. The SMD scale has previously demonstrated appropriate internal consistency, good convergent and criterion validity, and sufficient test-retest reliability [30]. Cronbach’s alpha in the present study indicated good reliability (α = 0.88).

2. Psychopathology

The survey also included measures of psychopathological symptoms (depression, anxiety, stress, and ADHD), impulsivity, and the big five personality traits (i.e., neuroticism, extraversion, conscientiousness, agreeableness and openness) to assure content validity as the PMPU-Q has previously been linked to wellbeing and personality [2,4,32]. The Depression-Anxiety-Stress Scale (DASS-21; [33]) consisted of 21 measures of depression, anxiety, and stress symptoms experienced in the previous two weeks rated on a scale ranging from 0 (symptom did not apply to me at all) to 3 (applied to me very much, or most of the time). Impulsivity was rated using a short form of the Barratt Impulsiveness Scale (BIS-15) [34,35]. The DASS-21 and BIS-15 are both well established and highly cited scales, consistently demonstrating strong validity, reliability, and excellent internal validity [33,35,36]. Measures of Attention Deficit Hyperactivity Disorder (ADHD) symptoms approved by the World Health Organisation were included in the survey [37] as ADHD has been associated with smartphone addiction [38].

3. Focus Group Procedure

Three independent focus groups were used including two trained facilitators (LH and DK), and were scheduled for approximately 90 min, and held in a quiet university research room. Focus group questions were designed to ask general questions about smartphone experiences (e.g., “Can anyone tell us your favourite/least favourite smartphone uses, and why?”) and to probe both beneficial and problematic aspects of smartphone experiences (e.g., “Can you tell us more about [experience]”). Eight prompt images were used on a PowerPoint slideshow to encourage discussion amongst participants, including images of smartphones used in different settings (e.g., on a train, in a library), and artistic depictions of smartphone use (e.g., cartoons and street art of smartphone use).

### 2.4. Analyses

#### 2.4.1. Quantitative Survey Analysis

The underlying structure of the PMPU-Q-R was assessed through exploratory factor analysis (EFA). This analysis was used to identify the latent constructs of smartphone experience underlying the variance in scores on measurements originally designed to measure mobile phone use. EFA analysis was conducted in IBM-SPSS with principle component analysis (PCA) and Direct Oblimin rotation, as recommended when establishing preliminary solutions [39,40].

CFA analysis was conducted to verify the factor structure of the variables. CFA was conducted in R package Lavaan using maximum likelihood estimators for CFA as they can effectively handle interactions between latent variables with multiple indicators [41,42]. To understand the fit of the model to the data, models were compared to threshold fit indices recommended by Hu and Bentler [43]. A model showing good fit to the data was expected to report CFI > 0.93, TLI > 0.93, RMSEA < 0.05 for very good fit, <0.084 for acceptable fit, and SRMR < 0.09.

The data set was randomly partitioned by a ratio 60:40 to run the factor analysis tests. Exploratory tests were performed on 60% of the data, and the proposed factor structure of the PMPUQ-R was examined on the remaining 40% of the data to test the proposed model on a second sample. This cross-validation approach is recommended to investigate the proposed model structure on independent datasets [44].

#### 2.4.2. Qualitative Thematic Analysis

Focus groups were audio recorded, and data were input into QSR-NVIVO, and analysed using thematic analysis [45] to explore smartphone experience. This involved familiarisation with the data by listening to and reading transcripts, generating initial codes for key features in the transcripts, searching for connections between codes (or themes), and reviewing and defining the identified themes based on the evidence presented across focus groups. Analysis was primarily conducted by Lydia Harkin. Themes were discussed amongst the wider team to assess the developing logic, in addition to discussing the apparent similarities and divergences within the qualitative and qualitative data.

### 2.5. Ethical Considerations

Participants provided informed consent to take part in this study. Only participants who agreed to be contacted for focus groups were invited for group interview. All contact information was stored securely, and after the study was completed, all identifying information was removed from the transcripts and destroyed. This study was given ethical approval by the Business, Law, and Social Sciences Ethics Committee at Nottingham Trent University and abided by the ethical codes of the British Psychological Society.

## 3. Results

Participants in the quantitative survey were 512 smartphone users. The sample had a mean age of 25.5 (range = 13–68 years), were primarily female (78.5%), university students (67%), and from the United Kingdom (91.8%). Table 2 demonstrates the breakdown of demographics represented in this sample.

### 3.1. Exploratory Factor Analysis

Firstly, 16 of the 17 items on the PMPU-Q-R correlated over 0.3 with at least one other item, indicating reasonable factorability [46,47]. Secondly, a Kaiser-Meyer-Olkin measure of sampling adequacy was 0.859, above the commonly recommended value of 0.6. Thirdly, Bartlett’s test of sphericity was significant (χ^2^ (120) = 1682.441, *p* < 0.001), indicating the present sample had a suitable size for factor analysis. Finally, the communalities of 16 of the 17 items were above 0.3, confirming that these 16 items shared common variance with other items. One item from the original ‘prohibited use’ scale did not share variance with the body of items, and was excluded from further analyses (“When using my mobile phone on public transport, I try not to talk too loud”). The EFA revealed a three-factor solution which explained 54% of the variance in scores. Cronbach’s alpha indicated the items consistently measured a closely related set of concepts (α = 0.86). All latent variables positively correlated with one another. For factors one and two, this correlation was moderate to strong (*R*^2^ = 0.436), whilst factor two correlated weakly with factor three (*R*^2^ = 0.172). However, factor three correlated very weakly with factor one (*R*^2^ = 0.043). Thus, the factors appeared to measure related, but distinct, concepts.

As Table 3 indicates, the pattern structure produced in the data did not correspond to the theoretical structure of the PMPU-Q-R. In line with the predefined PMPU-Q-R structure, all dependence items loaded highly together on one factor, with no cross-loadings onto factors two or three. The dependence factor explained 35% of variance in overall scores, and demonstrated high reliability (α = 0.89). A combination of seven items from the original prohibited and dangerous mobile phone use subscales loaded highly onto one factor, explaining 12% of the variance, suggesting the factor labels of ‘prohibited’ and ‘dangerous’ smartphone use could not be applied to the items within the scale for this population. On face value, the items contributing to this factor did not demonstrate an immediately apparent underlying theoretical property as demonstrated in Table 3. However, alpha scores indicated a high level of shared variance in scores (α = 0.77). Finally, two items from the dangerous subscale loaded highly onto factor three, explaining 8% of the score variance. A Cronbach’s alpha calculation is not meaningful for a two item factor, and therefore a Pearson correlation coefficient was calculated, showing a significant low to moderate correlation (*R*^2^ = 0.33, *p* < 0.001), indicating two distinct measures of a related concept. A review of these items revealed that they were the only PMPU-Q-R items to refer to driving behaviours: “I use my mobile phone while driving” and “I try to avoid using my mobile phone when driving on the motorway” (*R*^2^ = 0.43, *p* < 0.001).

This factor structure was tested using a CFA. As expected, all items showed significant positive factor loadings with standardised coefficients ranging from 0.482 to 0.805 (see Table 4). Additionally, modification indices were low (<50), which indicated that items corresponded to the proposed structure of the PMPUQ-R and did not covary too strongly with other items. The model showed an adequate fit to the data, with most of the indices of model fit falling within the acceptable values for the CFA model to fit the variance in scores (χ^2^ (101) = 190.424, *p* < 0.000, CFI = 0.927, TLI = 0.906, RMSEA = 0.062, SRMR = 0.054).

### 3.2. Construct Analyses

To shed light on the theoretical constructs underpinning the PMPUQ factor structure, Spearman’s correlations were calculated with the new factor structure and validated measures connected to problematic technology and smartphone behaviours. These were summed measures of smartphone and addictive social media use, psychopathology (i.e., depression, stress, anxiety; and ADHD), and impulsivity. These scales were correlated with the three-factor structure emerging from our survey. Only correlations which were significant and within the low to high range (0.2–1) are reported here. Notably, no significant correlations were found for the dangerous driving-related item scores.

An increase in dependence-related items strongly correlated with increased smartphone addiction symptoms (*R*^2^ = 0.67, *p* < 0.001), self-reported smartphone addiction (*R*^2^ = 0.66, *p* < 0.001), and moderately correlated with social media addiction symptoms (*R*^2^ = 0.44, *p* < 0.001). Additionally, dependence items weakly correlated with stress symptoms (*R*^2^ = 0.22, *p* < 0.001), and attention impulsivity (*R*^2^ = 0.26, *p* < 0.001).

Higher scores on the second, undefined factor strongly correlated with smartphone addiction symptom scores (*R*^2^ = 0.52, *p* < 0.001), and with social media addiction symptoms (*R*^2^ = 0.44, *p* < 0.001), self-reported smartphone addiction (*R*^2^ = 0.42, *p* < 0.001), attention impulsivity symptoms (*R*^2^ = 0.33, *p* < 0.001), motor impulsivity symptoms (*R*^2^ = 0.25, *p* < 0.001), and ADHD symptoms (*R*^2^ = 0.24, *p* < 0.001).

### 3.3. Thematic Analysis

Three focus groups were attended by a total of 21 individuals (11 females). Focus groups lasted an average of 96 min. Participant self-reported smartphone use ranged considerably, as highlighted in Table 2. The focus groups produced lively discussions about the integration of smartphones in participants’ lives. Thematic analysis of the focus group data revealed three distinct themes across perceptions of problematic smartphone use. These themes were smartphone dependence, dangerous driving, and antisocial smartphone use.

#### 3.3.1. Smartphone Dependence

Across all three focus groups, participants discussed the dependence their lives had on smartphones and applications. This theme incorporated both problematic and beneficial use. Many participants awoke with their smartphone alarms, read news or played games as they travelled to work or university, used email apps to support out-of-hours’ work, and to contact friends through social media. Thus, participants associated smartphones with many aspects of their day, much beyond the capabilities of traditional texting or calling. This was not perceived as necessarily problematic, and many participants emphasised that they benefitted from the integration of smartphones in day-to-day life.

P8: “My Smartphone is literally my life (laugh). Self-professed addict. I know the whole discussion has been generally negative, but erm, I still feel the positives outweigh any negatives that we get … (holds up phone) this is my calendar, this is how I communicate with everybody, how I organise my life, how I get up in the morning. It is my adult pacifier.”

*Focus group 1.*

Smartphone dependence was discussed as an act of balancing benefits with potentially problematic aspects. Daily smartphone use could lead to habitual checking behaviours and wasted time. Therefore, several participants made attempts to delete particular apps or groups of apps. For instance, across two focus groups, discussions around the utility of *Facebook* and other social media arose. These apps were perceived as ‘addictive’ because participants compulsively checked their social media, despite limited beneficial interactions or information. Most participants were unsuccessful in attempts to reduce time on smartphones due to the convenience and functionality of the devices. One participant had successfully stopped using all social media for 29 days prior to the focus group, and yet he found himself searching for other apps to use on his phone in replacement of his prior social media use. Therefore, the dependence on smartphones in daily life seemed to have a profound impact on the thoughts and actions of participants.

P5: “I have moulded my phone around me. My music, my pictures, everything. And I would probably feel lost without it.”

*Focus group 2.*

P1: (After quitting social media for 29 days) “So yeah my immediate reaction was like finding a replacement, not necessarily thinking oh I don’t have social media I need a replacement for it, but it was kind of what can I go on now? So it didn’t occur to me that I could just not go on my phone.”

*Focus group 2.*

#### 3.3.2. Dangerous Driving Behaviours

Of the problematic behaviours described by participants in the focus groups, driving whilst actively engaging with a smartphone was described as unequivocally problematic and dangerous. These types of actions were differentiated from some other potentially harmful smartphone uses. Participants were open to accepting smartphone use behaviours, such as crossing the road and walking, as an inevitable outcome of the proliferation of smartphone use. Discussions around emotionally dangerous behaviours, such as trolling online, were also perceived as a necessary evil of having constant exposure to the Internet. Thus, dangerous driving stood out distinctly as a problematic smartphone behaviour.

P2: “It (seeing people driving whilst on smartphones) infuriates you even when you see it and you know they are driving ridiculously.”

*Focus group 1.*

P5: “My best friend always does that (uses smartphone) in the car. I am like … you are on Instagram. (Name) what are you doing? You are going to kill me. I have to take her phone, I am holding your phone.”

*Focus group 3.*

P3: “Maybe you should learn how to cross the road safely while using your phone because that is what adult people do. It is like crossing the road with the red light on. We all do it. I do it at least, and I think I do it safely … I wouldn’t say don’t use it. I would say just be careful. Know how to use it.”

*Focus group 1.*

#### 3.3.3. Antisocial Smartphone Use

The antisocial properties of smartphone use emerged as a strong indicator of problematic behaviours, according to participants in this study. Participants were concerned that smartphone use often replaced face-to-face interactions. Groups of friends or couples were observed together in the ‘real world’, whilst seemingly disengaged from one another because they were using smartphones. This was perceived as indicative of poor social functioning. Similarly, several participants described feeling rejected or upset when their friends became distracted from their face-to-face conversations by their smartphones.

P2: “I feel personally … even if it’s in a group and someone starts taking their phone out I feel like a sting of rejection, so I’ll like sting them back. So I’m looking on my phone even if I’ve got no reason to. I’ll just try and find a reason to look on my phone and then hope that they see that I don’t need them either.”

*Focus Group 2.*

P6: “My partner absolutely drives me crazy on the phone. We go out for dinner … and he just sits there like that (mime sitting with phone in front). And that is all he does. And it is a nightmare going for dinner. And he never used to do it. All I can see is him like that (mime holding phone in front of face) constantly. It drives me mad.”

*Focus group 1.*

The antisocial properties of regular smartphone use seemed to govern and moderate participants’ behaviours. In particular, participants were concerned about how they would be perceived by others. Using smartphones too frequently or in dangerous or prohibited situations was perceived as embarrassing and reflective of their character. Many participants did not want to be viewed as someone who ‘needs’ their smartphone. For this reason, a few participants had enforced rules on how they, their partner, or friends were allowed to use their smartphones. For instance, several participants ‘banned’ smartphone use in restaurants during romantic meals, and others endeavoured not to use their smartphones while walking in the street.

P5: “I don’t want to be seen as someone who, like in a restaurant me and my boyfriend never have ours out … because I don’t want people to look at me and be like ‘oh god, she needs her phone out”

*Focus group 1.*

P9: “I hate the idea of being thought of as somebody who can’t put my phone down.”

*Focus group 3.*

## 4. Discussion

The present study aimed to investigate and validate an updated contemporary version of the original PMPU-Q, the PMPU-Q-R, using a rigorous and innovative convergent design. The PMPU-Q-R was tested to determine how many factors emerged from the scale, and how this corresponded with the theoretical underpinnings of the original PMPU-Q subscales [2]. Construct validity of the PMPU-Q-R items was investigated, alongside existing contemporary measures of problematic smartphone behaviours, and psychopathology. The quantitative data inquiry using an EFA revealed the pattern structure did not correspond to the expected and predefined structure of the PMPU-Q. Whilst the dependence factor was explained well by the data, a second factor was made up by a combination of items of the prohibited and dangerous subscales, suggesting the factor labels of ‘prohibited’ and ‘dangerous’ smartphone use could not be applied to the items within the scale for this sample. An explanation for this may be the rapid expansion and development of mobile technology since the development of the original PMPU-Q [2], which may have contributed to the results. Smartphone functionality has significantly increased in this time period, including the availability of high-quality satellite navigation and location-based augmented reality games (e.g., *Pokémon-GO*), changing the possible risks related to engaging in smartphone activities.

An increase in dependence-related items strongly correlated with increased self-reported smartphone addiction symptoms (measured via items considering excessive smartphone use as addictive disorder), and moderately correlated with social media addiction symptoms. This provides support for the construct validity of the dependence subscale. Previous research [31] has suggested that addictive smartphone use may be part of social media addiction. According to the pathway model of problematic mobile phone use [12], an addictive pattern of smartphone use is characterized by the use of specific applications, including calls and instant messaging. This definition could be extended by evidence from the focus group discussions; participants found that features, such as social media, emails, and games, contributed to increased feelings of dependence. This suggests that rather than being an addictive medium per se, mobile technologies including smartphones and tablets are media that enable the engagement in potentially addictive activities, including social media use. Similarly, it has been argued that individuals do not become addicted to the medium of the Internet per se, but to the activities they engage in on the Internet [48], such as gaming [49] or social media use [50]. With the advent and ubiquity of mobile technologies, this supposition appears particularly pertinent. Using social networking sites is a particularly popular activity on smartphones, with around 80% of social media used via mobile technologies [51], and around 75% of *Facebook* users access *Facebook* via their smartphones [52]. Consequently, social media use and smartphone use appear inherently intertwined [31], suggesting future research should pay additional attention to the forms and functions of specific smartphone use.

Additionally, dependence items weakly correlated with stress symptoms, and attention impulsivity. With regards to stress, it has been shown that the increased use of smartphones was related to general distress, anxiety and depression [13,53,54]. Further research [9] also highlighted that stress predicts addictive smartphone use. Individuals may use their smartphones to cope with everyday stressors (e.g., social situations, relationship problems), and using smartphones as coping mechanism can be considered dysfunctional, similar to using the Internet to cope with life problems [55], resembling symptoms traditionally associated with substance-related addictions [56].

Considering attention impulsivity, previous research [2] has shown that impulsivity was a strong predictor of problematic smartphone use, specifically with regards to the subscales urgency, lack of premeditation and lack of perseverance, which appear related to attention impulsivity [57]. Similarly, the present research found that higher scores on the dependence factor strongly correlated with ADHD symptoms, which is in line with previous research [58] in children. Alternatively, it has been suggested that particular types of activities engaged in on smartphones, e.g., gaming, may lead to the development of ADHD symptoms, suggesting future research may be necessary to disentangle the differential impact of specific smartphone application use on possible dependence.

In addition to this, the thematic analysis applied in the present research revealed that there appears to be a strong awareness that using smartphones whilst driving can be dangerous both for the self and for others, which corroborates the quantitative data regarding the PMPU-Q-R dangerous driving factor. This suggests the driving factor is a valid and reliable factor that contributes to explaining contemporary problematic smartphone use, and should therefore be retained in future analyses of problematic smartphone use.

Findings from the qualitative and quantitative analysis suggested that dangerous driving stands out as a distinct form of problematic smartphone behaviour. This corroborates the US Department of Transportation’s report showing smartphone use can distract pedestrians and drivers, leading to potential collisions [28]. In 2014, over 3000 individuals died in the US as a consequence of being distracted while driving, leading the US National Highway Traffic Safety Administration (NHTSA) to issue voluntary guidelines for smartphone developers, which aim to restrict the functions of smartphones being used by a driver. A recent report by the American Automobile Association Foundation found that using smartphones, including Apple’s voice control system Siri, is very dangerous in the context of driving as it leads to cognitive distraction [59].

The thematic analysis of the focus group data revealed three distinct themes across perceptions of problematic smartphone use, namely smartphone dependence, dangerous driving, and antisocial smartphone use. With regards to the first theme, smartphone dependence, the thematic analysis indicated smartphones are essential elements of individuals’ lives as they are being used for their many functions, going beyond phone calls and texting, including other entertainment functions (e.g., music, pictures), as well as organisational functions (i.e., calendar, alarm). Participants perceived particular smartphone applications as being potentially addictive, including social media, which they were checking compulsively, although there were limited advantages of doing so. Participants found it difficult to reduce the time they spent on their smartphones as these were perceived to be very convenient and functional.

Antisocial smartphone use emerged as a key problematic behaviour, as evidenced through the thematic analysis. Importantly, when looking at the prohibited use items of the PMPU-Q-R, clear links between the qualitative and quantitative analyses emerged. Indeed, most of the prohibited items refer to situations where using smartphones is banned, implying that scoring highly on these items depicts engaging in antisocial behaviours. Beside prohibited use per se, this was a key observation as some individuals use their smartphones in social contexts, which may similarly appear as antisocial, and can consequently impact negatively on their overall social functioning, both in terms of the quality of interaction with others, and with regards to perceptions of rudeness and rejection in interpersonal contexts. Along the same lines, recent research focused on the “phubbing” phenomenon, defined as the act of snubbing someone in a social setting by using one’s phone instead of interacting, and research has shown that such types of antisocial smartphone use are linked to lack of self-control [60] and lower relationship satisfaction among romantic partners [61].

In the current study, participants discussed how they disapproved of friends or couples disengaging from one another whilst engaging with their smartphones, leading to feeling devalued. Similar situations and behaviours have been observed in the context of young people disconnecting from their offline contacts for the sake of connecting online, which has been linked to a preference for online social interaction [62]. This was tied to an awareness of public perceptions on the individuals’ smartphone use, often leading to behavioural change in terms of limiting use in particular situations and contexts, as found in the present research.

With regards to the integration of both methods using the adopted convergent design, the findings confirm that a combination of prohibited and dangerous items from the PMPU-Q-R may be explained by antisocial smartphone use. Further research is necessary to inquire about motivations for smartphone use, as well as the norms of smartphone use in public, given that stigma and public perceptions appear to significantly contribute to how smartphone use is perceived by the users regarding being prohibited or dangerous. The focus group data analysis furthermore revealed that public perceptions may lead to behavioural change in terms of how individuals engage with their smartphones, emphasising the need to assess problematic smartphone use within its sociocultural context, bearing in mind the cultural and behavioural norms associated with smartphone use. Using anthropological and cultural studies may aid our understanding and study of the impacts of technology use as it has been shown to be particularly insightful in the study of specific technology use, such as gaming, given it allows for an assessment of the behavioural norms and practices surrounding a concrete behaviour [63,64]. The individual’s context is a significant factor that can mark the dividing line between problematic smartphone use and potential smartphone “addiction”, and the smartphone use context can gain particular importance for users, depending on their life situation (i.e., the meaning they attach to their smartphone) and smartphone use preferences (i.e., particular types of apps used and activities engaged in). Moreover, the cultural context is significant because it embeds the smartphone user in a community with shared beliefs and practices, endowing their use with particular meaning as well as possible stigma. The context of the individual, the specific smartphone use and the smartphone use environment, as well as the broader framework of the respective culture the user is situated in are relevant in the study of problematic smartphone use and are therefore recommended to be used in the context of future smartphone use research [10].

Regarding the methodology that has been utilised in the present research, mixed methods have been employed, integrating quantitative with qualitative techniques. There was an existing body of theory to draw on [12], but it needed updating, so mixed methods allowed us to combine previous theory with present experiential understanding. The mixing of quantitative with qualitative methods is a challenging endeavour, particularly as these methods can be understood as separate scientific paradigms. One could claim these methods are incommensurate as their unit of analysis (i.e., words versus numbers), their epistemological position (i.e., knowledge derived from meaning versus behaviours), and their source of scientific knowledge (i.e., induction versus deduction) are inherently incompatible. Accordingly, the adherence to a single method could be seen as the epitome of normal science, indicating a scientific revolution is necessary to integrate the seemingly incongruous positions of quantitative and qualitative research. This integration overcomes the limitations of a single methodology, i.e., its inevitable incompleteness. The usage of mixed methods, on the other hand, allows for the corroboration, elaboration, and complementation of findings [65]. The present study corroborated the PMPU-Q-R structure in terms of the dependence factor, and suggested that dangerous driving is a distinct factor that needs considering when studying problematic smartphone use. Moreover, the qualitative element of this research complemented the quantitative findings with regards to the combination of the dangerous and prohibited factor by elaborating on how antisocial use and public perceptions may contribute to individual perceptions of norms surrounding smartphone use in different contexts.

## 5. Conclusions 

The updated version of the PMPU-Q, the PMPU-Q-R, is a valid and reliable tool for measuring contemporary smartphone use and problems associated with this use, concerning dependence, antisocial use and dangerous driving. Future research is encouraged to discern user motivations and perceptions of usage norms and meanings applied to smartphone use to delineate the impact social stigma may have on smartphone use. The convergent design used in the present study appeared to offer a corroborative and complementary perspective on contemporary knowledge of problematic smartphone use, and has expanded the knowledge base in the field of behavioural technological addictions. Taken together, contemporary smartphone use may become problematic if engaged in excessively. Nonetheless, contemporary smartphone users often actively seek to modify their behaviours when they are being perceived as problematic, suggesting users appear aware and conscious of their usage patterns, which may limit the extent of problematic use.

## Figures and Tables

**Table 1:** Items added to the PMPUQ.

Item Question
I use my mobile phone whilst crossing the road.
I have found myself in risky situations because I have used my mobile phone whilst walking.

**Table 2:** Survey and Focus Group Participant Demographics.

Participant Demographics	Survey Participants *N* (%) *	FG Participants *N* (%)
Gender		
Male	401 (20.9)	10 (47.6)
Female	107 (78.3)	11 (52.4)
Prefer not to say	3 (0.6)	0
Country of Origin		
United Kingdom	470 (91.8)	21 (100)
USA	10 (2)	
Ireland	4 (0.8)	
Other	49 (6.4)	
Level of Education		
No formal qualifications	4 (0.8)	0
GCSEs	13 (2.5)	0
Further education	286 (55.9)	0
Vocational qualification	8 (1.6)	10 (47.6)
Higher education	122 (23.8)	2 (9.5)
Postgraduate degree	79 (15.4)	9 (42.9)
Self-reported calls per day		
0–1 year	262 (51.2)	
2–5 years	214 (41.8)	
5–10 years	27 (5.3)	
>10 years	8 (1.6)	
Self-reported texts per day		
0–5	50 (9.8)	1 (4.8)
5–10	64 (12.5)	6 (28.6)
10–20	82 (16)	4 (19)
20–30	79 (15.4)	2 (9.5)
30–40	39 (7.6)	3 (14.3)
>40	198 (38.7)	4 (19)
Self-reported time spent on phone p/day		
<30 min	10 (2)	0
30 min–1 h	34 (6.6)	4 (19)
1–2 h	134 (26.2)	8 (38)
3–5 h	219 (42.8)	7 (33.3)
5–10 h	92 (18)	2 (9.5)
>10 h	22 (4.3)	0

* Note. Rounding may have led to percentages that do not equal 100.

**Table 3:** Factor loadings for the PMPUQ-R items.

Original Item Subscale		Factor Loading		
		1	2	3
Dependence	I can easily live without my mobile phone *	0.869		
Dependence	I feel lost without my mobile phone	0.844		
Dependence	It is hard for me to turn my mobile phone off	0.769		
Dependence	It is easy for me to spend all day not using my mobile phone	0.747		
Dependence	I get irritated when I am forced to turn my mobile phone off	0.699		
Dependence	I don’t attach a lot of importance to my mobile phone *	0.694		
Dependence	Is it hard for me not to use my mobile phone when I feel like it	0.495		
Prohibited use	I don’t use my mobile phone when it is completely forbidden to use it *		0.701	
Prohibited use	I don’t use my mobile phone in a library, cinema, or hospital *		0.700	
Prohibited use	I use my mobile phone where it is forbidden to do so		0.673	
Danger	I have found myself in risky situations because I have used my mobile phone whilst walking		0.583	
Danger	I use my mobile phone whilst crossing the road		0.574	
Prohibited use	I try to avoid using my mobile phone where people need silence *		0.568	
Danger	I use my mobile phone in situations that would qualify as dangerous		0.563	
Danger	I use my mobile phone while driving			0.758
Danger	I try to avoid using my mobile phone when driving on the motorway			0.751

* Reversed item.

**Table 4:** Beta coefficients and significance values of PMPUQ-R factor loadings.

Latent Factor	Item	B	Standard Error	Beta (Standardised)	Sig
Dependence	x1	1.000		0.504	**
Dependence	x2	0.941	0.278	0.485	***
Dependence	x3	1.000		0.661	***
Dependence	x4	0.847	0.148	0.474	***
Dependence	x5	1.053	0.136	0.627	***
Dependence	x6	0.763	0.13	0.483	***
Dependence	x7	0.978	0.141	0.599	***
Factor 2	x8	0.895	0.126	0.602	***
Factor 2	x9	0.745	0.124	0.482	***
Factor 2	x10	1.000		0.79	***
Factor 2	x11	0.570	0.07	0.568	***
Factor 2	x12	0.797	0.075	0.72	***
Factor 2	x13	0.774	0.073	0.714	***
Factor 2	x14	0.954	0.075	0.805	***
Danger	x15	0.839	0.08	0.714	***
Danger	x16	0.849	0.075	0.752	***

*** *p* < 0.001; ** *p* = 0.001.
